# A Weibull process monitoring with AEWMA control chart: an application to breaking strength of the fibrous composite

**DOI:** 10.1038/s41598-023-47159-9

**Published:** 2023-11-14

**Authors:** Muhammad Atif Sarwar, Muhammad Noor-ul-Amin, Imad Khan, Emad A. A. Ismail, Wojciech Sumelka, Muhammad Nabi

**Affiliations:** 1 COMSATS University Islamabad-Lahore Campus, Lahore, Pakistan; 2 Department of Statistics, Abdul Wali Khan University Mardan, Mardan, 23200 Pakistan; 3 Department of Quantitative Analysis, College of Business Administration, King Saud University, P.O. Box 71115, Riyadh 11587, Saudi Arabia; 4 Institute of Structural Analysis, Poznan University of Technology, Poznan, Poland; 5 Khost Mechanics Institute, Khowst, Afghanistan

**Keywords:** Energy science and technology, Engineering, Mathematics and computing

## Abstract

In recent times, there has been a growing focus among researchers on memory-based control charts. The Exponentially Weighted Moving Average (EWMA) and Cumulative Sum (CUSUM) charts and the adaptive control charting approaches got the attention. Control charts are commonly employed to oversee processes, assuming the monitored variable follows a normal distribution. However, it's worth noting that this assumption does not hold true in many real-world situations. The use of the algebraic expression for normalization, which can be used for all kinds of skewed distributions with a closed-form distribution function, using the proposed continuous function to adapt a smoothing constant, motivates this study. In the present manuscript, we design an EWMA statistic-based adaptive control chart to monitor the irregular variations in the mean of two parametric Weibull distribution and use Hasting approximation for normalization. The adaptive control charts are used to update the smoothing constant according to the estimated shift. Here we use the proposed continuous function to adapt the smoothing constant. The average run length and standard deviation of run length are calculated under different parameter settings. The effectiveness of the proposed chart is argued in terms of ARLs over the considered EWMA chart through Monte-Carlo (MC) simulation method. The proposed chart is examined, followed by a real data set to demonstrate the design and application procedures.

## Introduction

The quality control charts (CCs) got special attention, in the literature on statistical process control (SPC). Due to their ease, adequacy, and efficiency to track deviations in the process. The fundamental part of SPC is identifying and monitoring the special cause variations in a production process. This identification and monitoring help improve the product's quality and the process's competence^1^. The memory-less and memory-based categories of variable CCs can be distinguished. CCs that monitor process parameters solely based on current sample data are considered to as memory-less CCs. Memory-based CCs are those that make use of both historical and current statistics data. When we use prior sample information it improves the responsiveness. To detect the small magnitude of the shifts, one can use memory based CC﻿s. The CCs which was introduced by W. A. Shewhart in 1924 is a fundamental device in quality improvement. Subsequently, the adaptive method for uplifting CCs is identified by﻿^2^. The Shewhart ﻿CCs, which are generally used for monitoring and improving the process, are examples of memory-less CCs. On the other hand, cumulative sum (CUSUM) and exponentially weighted moving average (EWMA) CCs are examples of memory-based ﻿CCs. The EWMA ﻿CC was introduced by^3^ to monitor the mean of a normally distributed process. In comparison, the CUSUM ﻿CC was presented by^4^ to observe the deviations in the process. The EWMA statistic attracts many researchers and engineers because of its ability to distinguish the small magnitude of the shift in the process parameter(s) early. For additional subtleties, the reader may consider^5, 6^.

Although these CCs are important when the shift size is known somewhat early or when the quality expert is interested in designing a CC for a specific shift. In most cases, the size of the shift may not be known in advance. In what follows, the quality specialists began dealing with improving adaptive control charting plans that provide further enhanced protection against various sizes of shifts. An adaptive EWMA (AEWMA) ﻿CC that combines memory-less Shewhart CC and memory-based EWMA charts smoothly. The fundamental belief is to choose the past observation weight, as demonstrated by the size of the error $({e}_{t}={y}_{t}-{x}_{t-1})$, to recognize the different sizes of shifts with reducing the inertia problem. A similar philosophy was trailed by^7^, and the authors proposed an AEWMA ﻿CC for monitoring the process's mean, which follows the normal distribution. The authors used score functions in developing their suggested AEWMA ﻿CC as they are good at combining the highlights of Shewhart and EWMA CCs. The authors exhibited that the proposed AEWMA charts perform well when contrasted with the conventional Shewhart, Shewhart EWMA, optimal CUSUM, and optimal EWMA charts while noticing the diverse size of shifts in the process mean. For instance, Yoon et al.^8^ presented the AEWMA-*X* ﻿CCs by using Kalman recursive average and reported that the proposed chart is more efficient than the EWMA-*X* ﻿CC when the process mean is shifted. Abbas et al.^9^ presented the non parametric controlc chart by using the progressive mean. Shu et al.^10^ presented ACUSUM ﻿CC with two-dimensional Markov chain models. Jiang et al.^11^ suggested using the EWMA shift estimator to create an ACUSUM CC. Inspired by the notion of likelihood ratio testing, they have put forth a linear weighted function that achieves better detection performance. Su et al.^12^ examined how well the AEWMA CC performed at identifying linear drifts.﻿ Zamam et al.^13^ presented AEWMA ﻿CC with the Hample function to monitor the process mean. Abbas et al.^14^ proposed poisson adaptive EWMA ﻿CC for the mean. Riaz et al.^15^ discussed AEWMA ﻿CC for monitoring the coefficient of variation ﻿CC under ranked set sampling. An adaptive algorithm is used by^16^ to analyze the dynamic monitoring system of the break provisions in energy storing systems or voltage contrast deficiencies on unambiguous grounds. The proposed AEWMA chart based on the proposed continuous function to monitor process mean was performed capably in recognizing a wide range of diminishing and expanding process mean shift sizes^17^. Noor-ul-Amin et al. ^18^ proposed the AEWMA ﻿CC based on the continuous function for multivariate cases.

Generally, CCs are planned under the presumption that the variable under study came from the normal distribution. Practically, this may not be valid in every case. The design of a CC under the normality assumption may deceive manufacturing engineers into noticing a process shift. When data is assumed to follow a normal distribution, any deviations from this assumption may lead to false alarms, indicating the presence of special causes when there are none. This can result in unnecessary investigations and adjustments, increasing costs and inefficiencies. As per^19^, the usually utilized individual ﻿CCs do not produce great outcomes since the data not gathered in subgroups might be exceptionally skewed; thus, other approaches should be used for adequacy. For monitoring the non-normal process, efficient sequential-based schemes have been presented by^20^. The same problem can be seen in^21, 22^. In the present manuscript, we consider a positive skewed two-parameter Weibull distribution. Many real-world processes do not follow a perfect normal distribution. The Weibull distribution can handle skewed and non-normal data better than some other distributions. When dealing with such data, ﻿CCs based on the Weibull distribution can provide more accurate insights into process behavior. The Weibull distribution is very flexible and can model a wide range of shapes, including exponential, normal, and even skewed distributions. This flexibility allows it to adapt to various types of data, making it useful in different industries and applications. Weibull distribution is commonly used in reliability analysis to predict the failure rates of products and systems. By incorporating this distribution into CCs, companies can monitor the reliability of their processes over time. This is especially important in industries where the reliability of products is critical, such as aerospace and automotive. The Weibull distribution is viewed as a prevalent model for skewed data and is extensively used to portray material-breaking characteristics^23, 24^, in reliability to address the lifetimes of electronic parts and products. Here, we utilize the transformation given by^25^ to advance an AEWMA CC. We first gauge the mean shift utilizing the EWMA statistic with the assistance of an estimator. Afterward, we calculate the value of the smoothing constant using the proposed continuous function based on the magnitude of shift size. The run length (RL) profiles of the proposed AEWMA (abbreviated as AEWMA-I) CC are figured with the assistance of the MC simulation method. The AEWMA-I CC is compared with the EWMA chart as far as the ARL. The AEWMA-I chart outclasses the EWMA chart.

The rest of the manuscript is ordered as given. The detailed design of EWMA and proposed new AEWMA-I CC for Weibull transformed data using Hastings’s approximation for effectively observing changes in the process mean is given in "Design of the proposed CCs for Weibull distribution". In “Performance evaluation”, the RL profile computation is presented. In "Performance comparison", evaluation of the proposed method is done. An illustrative example of using an industrial dataset to clarify the applicability and supremacy of the sug-gested CC is presented in "Illustrative example". The main points about the results are given in "Main findings". The conclusions of the article are given in “Conclusion”.

## Design of the proposed control charts for Weibull distribution

In this segment, we design new EWMA and AEWMA-I ﻿CCs for observing irregular changes in the mean of a Weibull process. Let *U* denote a Weibull random variable. We note $U\sim W\left(\eta ,\theta \right)$, where $\eta$ and $\theta$ respectively, denote the scale and the shape parameters ($\eta$*,* $\theta$ > 0). The following cumulative distribution function can characterize this distribution:

1 $$H\left(u\right)=P\left(U\le u\right)=1-exp\left[-{\left(\frac{u}{\eta }\right)}^{\theta }\right] ,u>0 \,and\, \eta ,\theta \in \left(0, \infty \right).$$

The Exponential distribution is a reduced model of Weibull distribution for $\theta$ is equal to 1.

Assuming the random sample of size $n$ is taken from the sequence $\left\{{U}_{t}\right\}$, which follows a two-parameter Weibull distribution at time $t$ as $\left\{{U}_{1t},{U}_{2t},\dots ,{U}_{nt},\right\}$; ${U}_{it}$ gives the *i*th observation in the tth sample with $i={1,2},\dots ,n,$ and $t\ge 1$.

### Hastings’s approximation to normal

The algebraic approximation proposed by^25^ converts the random variable of skewed distribution to the standard normal variate Z using its distribution function.

2 $$Z=-\left(x-\frac{{c}_{0}+{c}_{1}x+{c}_{2}{x}^{2}}{1+{d}_{1}x+{d}_{2}{x}^{2}+{d}_{3}{x}^{3}}\right), \forall H\left(u\right)\in \left(0, 0.5\right],$$

3 $$Z=+\left(x-\frac{{c}_{0}+{c}_{1}x+{c}_{2}{x}^{2}}{1+{d}_{1}x+{d}_{2}{x}^{2}+{d}_{3}{x}^{3}}\right), \forall H\left(u\right)\in \left(0.5, 1.0\right).$$

where:

$$x = \sqrt {\ln \left( {\frac{1}{{\left( {H(u)} \right)^{2} }}} \right)} ,\quad \forall H(u) \in (0,0.5],$$

$$x = \sqrt {\ln \left( {\frac{1}{{\left( {1 - H(u)} \right)^{2} }}} \right)} ,\quad \forall H(u) \in (0.5,\quad 1.0),$$

$${c}_{0}=2.515517, {c}_{1}=0.802853, {c}_{2}=0.010328,$$

$${d}_{1}=1.432788, {d}_{2}=0.189269, {d}_{3}=0.001308,$$

now,

4 $${V}_{t}=\sqrt{n}{\overline{Z} }_{t}.$$

For the in-control process, ${V}_{t}$ is given as, i.e., ${V}_{t}\sim N\left({0,1}\right)$.

### The EWMA control chart for Weibull distribution

The plotting statistic ${E}_{t}$ of the EWMA ﻿CC suggested by^3^ based on the sequence $\left\{{V}_{t}\right\}$ is

5 $${E}_{t}=\lambda {V}_{t}+\left(1-\lambda \right){E}_{t-1}, \mathrm{t }\in {Z}^{+},$$

where ${E}_{0}$ = 0 is the initial value and $\lambda \in ({0,1}]$. The mean and variance of ${E}_{t}$ is

$$E\left({E}_{t}\right)=0,$$

$$Var\left({E}_{t}\right)=\frac{\lambda }{2-\lambda }\left[1-{\left(1-\lambda \right)}^{2t}\right].$$

The asymptotic variance for $t\to \infty$ is

$$Var\left({E}_{t}\right)=\frac{\lambda }{2-\lambda },$$

therefore, the control limits of the EWMA CC are as follows

$$LCL/UCL=\mp H\sqrt{\frac{\lambda }{2-\lambda }} ,$$

where *H* is the EWMA CC's coefficient of control limit for the given in-control ARL. The value of *H* (*H* > 0) is a threshold for a given n and λ. In order to guarantee the in-control ARL optimum sensitivity of the EWMA CC statistic |E_t | at a predetermined fixed level (let’s say ARL_0_), the value of *H* is calculated. where *H* is the coefficient of control limit of the EWMA CC for specified in-control ARL. The MC method is used for simulation study. To get the mean and the standard deviation of RL of the EWMA chart, the sample sizes *n* = 3, 4, and 5 are used. We have fixed in-control ARL (ARL_0_ = 370) with $\phi =$ 0.15 and 0.20 for some fixed values of shift sizes. The RL profiles of the EWMA ﻿CC are presented in Tables 1 and 2.

**Table 1:** The results of the EWMA chart when $\phi$ = 0.15, *L* = 2.7995, and subgroup size *n* = 3, 4, 5.

Scale shift (${\eta }_{1}/{\eta }_{0}$)	*N*	Shape parameter = $\theta$							
		0.5	1	1.5	2	2.5	3	3.5	4
		ARL (SDRL)	ARL (SDRL)	ARL (SDRL)	ARL (SDRL)	ARL (SDRL)	ARL (SDRL)	ARL (SDRL)	ARL (SDRL)
1	3	369.56 (361.39)	369.35 (364.24)	370.36 (363.66)	370.80 (365.80)	370.45 (365.33)	370.43 (365.08)	370.71 (366.86)	370.17 (364.31)
	4	369.21 (365.62)	369.65 (364.53)	370.15 (361.41)	369.61 (362.77)	369.84 (365.23)	369.43 (364.55)	370.08 (363.66)	369.90 (365.99)
	5	369.15 (362.01)	369.93 (365.70)	369.76 (364.20)	369.10 (362.49)	369.22 (364.70)	369.55 (364.82)	370.44 (362.98)	370.12 (363.13)
1.1	3	275.41 (269.88)	167.60 (161.18)	100.91 (94.65)	64.26 (57.72)	43.76 (37.79)	31.33 (25.64)	23.76 (18.32)	18.72 (13.76)
	4	260.28 (254.95)	146.80 (140.73)	84.05 (77.99)	51.54 (45.16)	34.58 (28.69)	24.90 (19.46)	18.90 (13.83)	14.93 (10.18)
	5	247.77 (241.66)	130.24 (125.07)	71.44 (64.67)	43.11 (36.60)	28.71 (23.01)	20.80 (15.51)	15.72 (10.81)	12.60 (8.09)
1.2	3	176.15 (168.81)	69.19 (62.56)	34.16 (28.25)	20.28 (15.09)	13.68 (9.20)	10.17 (6.19)	8.02 (4.52)	6.55 (3.39)
	4	155.05 (149.17)	55.75 (49.40)	27.15 (21.5)	16.10 (11.27)	11.12 (6.90)	8.34 (4.69)	6.60 (3.40)	5.46 (2.59)
	5	136.60 (130.51)	46.77 (40.69)	22.48 (17.04)	13.57 (8.98)	9.45 (5.44)	7.11 (3.70)	5.73 (2.72)	4.77 (2.11)
1.4	3	79.27 (72.74)	23.41 (18.12)	11.55 (7.31)	7.40 (4.05)	5.34 (2.57)	4.17 (1.85)	3.41 (1.42)	2.88 (1.14)
	4	64.58 (57.47)	18.62 (13.42)	9.43 (5.49)	6.13 (3.06)	4.49 (1.99)	3.53 (1.43)	2.93 (1.11)	2.50 (0.90)
	5	53.74 (47.14)	15.63 (10.78)	8.04 (4.40)	5.34 (2.49)	3.94 (1.61)	3.14 (1.18)	2.62 (0.93)	2.25 (0.76)
1.8	3	29.79 (23.94)	9.07 (5.31)	4.99 (2.37)	3.42 (1.42)	2.58 (1.00)	2.07 (0.77)	1.72 (0.66)	1.47 (0.57)
	4	23.66 (18.17)	7.47 (4.01)	4.24 (1.83)	2.94 (1.11)	2.25 (0.80)	1.82 (0.65)	1.51 (0.57)	1.30 (0.47)
	5	19.63 (14.35)	6.44 (3.20)	3.72 (1.49)	2.62 (0.93)	2.03 (0.69)	1.64 (0.59)	1.36 (0.50)	1.18 (0.39)
2.5	3	13.64 (9.10)	4.75 (2.21)	2.80 (1.10)	1.98 (0.74)	1.52 (0.58)	1.24 (0.44)	1.11 (0.31)	1.04 (0.20)
	4	11.04 (6.76)	4.01 (1.70)	2.43 (0.88)	1.74 (0.63)	1.33 (0.49)	1.12 (0.33)	1.04 (0.19)	1.01 (0.10)
	5	9.35 (5.38)	3.55 (1.40)	2.19 (0.74)	1.57 (0.58)	1.21 (0.41)	1.06 (0.24)	1.01 (0.11)	1.00 (0.05)
4.5	3	6.27 (3.21)	2.52 (0.97)	1.54 (0.59)	1.15 (0.37)	1.04 (0.19)	1.01 (0.09)	1.00 (0.03)	1.00 (0.02)
	4	5.25 (2.45)	2.19 (0.78)	1.36 (0.51)	1.06 (0.25)	1.01 (0.09)	1.00 (0.03)	1.00 (0.01)	1.00 (0.00)
	5	4.58 (2.00)	1.98 (0.68)	1.23 (0.43)	1.03 (0.16)	1.00 (0.04)	1.00 (0.01)	1.00 (0.01)	1.00 (0.00)

**Table 2:** Relative investigation of RL profile at ARL_0_ = 370 and $\phi$ = 0.15 at different sample sizes.

Scale shift (${\eta }_{1}/{\eta }_{0}$)	*n*	Shape parameter = $\theta$							
		0.5		1		2		3	
		EWMA	AEWMA-I	EWMA	AEWMA-I	EWMA	AEWMA-I	EWMA	AEWMA-I
		ARL (SDRL)	ARL (SDRL)	ARL (SDRL)	ARL (SDRL)	ARL (SDRL)	ARL (SDRL)	ARL (SDRL)	ARL (SDRL)
1.1	3	275.41 (269.88)	211.47 (186.52)	167.60 (161.18)	110.23 (86.41)	64.26 (57.72)	46.82 (33.59)	31.33 (25.64)	25.64 (18.40)
	4	260.28 (254.95)	190.74 (165.69)	146.80 (140.73)	94.96 (72.00)	51.54 (45.16)	38.48 (27.41)	24.90 (19.46)	20.83 (14.92)
	5	247.77 (241.66)	175.63 (148.20)	130.24 (125.07)	84.07 (61.93)	43.11 (36.60)	33.17 (23.44)	20.80 (15.51)	17.52 (12.48)
1.2	3	176.15 (168.81)	116.27 (91.46)	69.19 (62.56)	49.48 (35.63)	20.28 (15.09)	17.29 (12.54)	10.17 (6.19)	8.64 (6.07)
	4	155.05 (149.17)	99.87 (76.36)	55.75 (49.40)	41.38 (29.26)	16.10 (11.27)	13.82 (9.82)	8.34 (4.69)	7.01 (4.70)
	5	136.60 (130.51)	88.57 (66.03)	46.77 (40.69)	35.37 (25.08)	13.57 (8.98)	11.76 (8.20)	7.11 (3.70)	5.88 (3.81)
1.4	3	79.27 (72.74)	55.23 (39.91)	23.41 (18.12)	19.76 (14.28)	7.40 (4.05)	6.14 (4.13)	4.17 (1.85)	3.18 (2.01)
	4	64.58 (57.47)	46.18 (32.95)	18.62 (13.42)	15.91 (11.36)	6.13 (3.06)	4.97 (3.19)	3.53 (1.43)	2.61 (1.60)
	5	53.74 (47.14)	40.00 (28.26)	15.63 (10.78)	13.38 (9.45)	5.34 (2.49)	4.22 (2.61)	3.14 (1.18)	2.23 (1.34)
1.8	3	29.79 (23.94)	24.52 (17.63)	9.07 (5.31)	7.65 (5.30)	3.42 (1.42)	2.49 (1.57)	2.07 (0.77)	1.40 (0.73)
	4	23.66 (18.17)	19.96 (14.23)	7.47 (4.01)	6.23 (4.11)	2.94 (1.11)	2.06 (1.24)	1.82 (0.65)	1.23 (0.52)
	5	19.63 (14.35)	16.87 (11.94)	6.44 (3.20)	5.27 (3.37)	2.62 (0.93)	1.77 (1.03)	1.64 (0.59)	1.12 (0.38)
2.5	3	13.64 (9.10)	11.73 (8.37)	4.75 (2.21)	3.70 (2.36)	1.98 (0.74)	1.34 (0.66)	1.24 (0.44)	1.04 (0.20)
	4	11.04 (6.76)	9.40 (6.60)	4.01 (1.70)	3.03 (1.86)	1.74 (0.63)	1.18 (0.47)	1.12 (0.33)	1.01 (0.11)
	5	9.35 (5.38)	7.95 (5.34)	3.55 (1.40)	2.60 (1.56)	1.57 (0.58)	1.10 (0.34)	1.06 (0.24)	1.00 (0.06)
4.5	3	6.27 (3.21)	5.08 (3.35)	2.52 (0.97)	1.72 (1.01)	1.15 (0.37)	1.02 (0.14)	1.01 (0.09)	1.00 (0.02)
	4	5.25 (2.45)	4.14 (2.59)	2.19 (0.78)	1.45 (0.77)	1.06 (0.25)	1.00 (0.07)	1.00 (0.03)	1.00 (0.00)
	5	4.58 (2.00)	3.54 (2.15)	1.98 (0.68)	1.29 (0.60)	1.03 (0.16)	1.00 (0.03)	1.00 (0.01)	1.00 (0.00)

### Design of the proposed AEWMA control chart for Weibull distribution

Practically speaking, the magnitude of shift size is not known ahead of time. So here we consider the estimator proposed by^26^ to estimate the magnitude of shift size as

6 $${{\widehat{\delta }}^{*}}_{t}= \phi {V}_{t}+\left(1-\phi \right){{\widehat{\delta }}^{*}}_{t-1},$$

7 $${{\widehat{\delta }}_{t}}^{**}= \frac{{{\widehat{\delta }}_{t}}^{*}}{1-{\left(1-\phi \right)}^{t}}$$

where $$\phi \in ({0,1}]$$ , $${{\widehat{\delta }}_{0}}^{*}=0$$. Consider $${\stackrel{\smile}{\delta }}_{t}=\left|{{\widehat{\delta }}_{t}}^{**}\right|$$ when estimating the magnitude of shift size. Using the sequence $$\left\{{V}_{t}\right\}$$, the plotting statistic of the AEWMA-I CC for observing the process mean is given by

8 $${F}_{t}= g\left({\stackrel{\smile}{\delta }}_{t}\right){V}_{t}+\left(1-g\left({\stackrel{\smile}{\delta }}_{t}\right)\right){F}_{t-1},$$

where ${F}_{0}=0$ and $g\left({\stackrel{\smile}{\delta }}_{t}\right)\in ({0,1}]$ such that

9 $$g({\stackrel{\smile}{\delta }}_{t})=\left\{\begin{array}{l}\begin{array}{l}\frac{1}{24\left[1+{\left({\stackrel{\smile}{\delta }}_{t}\right)}^{-2}\right]}\quad \forall {\stackrel{\smile}{\delta }}_{t}\in (0, 1.0]\\ \frac{1}{19\left[1+{\left({\stackrel{\smile}{\delta }}_{t}\right)}^{-1}\right]} \quad \forall {\stackrel{\smile}{\delta }}_{t}\in (1.0, 2.7]\end{array}\\ 1\quad \forall {\stackrel{\smile}{\delta }}_{t}\in \left(2.7, \infty \right).\end{array}\right.$$

Drawing inspiration from binary logistic regression. Where the response function is constrained within the range of 0 to 1, we conducted an empirical exploration. This is done byexperimenting with different functions (such as logarithmic, exponential, and others) along with different constants. Our aim was to fine-tune the traditional MEWMA scheme to achieve near-optimal performance in detecting shifts within the predefined ranges. Since the design of the function plays a crucial role in managing the false alarm rate, it's important to note that the standard deviation of the run-length is closely tied to this false alarm rate. This is why our proposed AMEWMA exhibits a more compact run-length profile when compared to competing CCs.

The constants used in the function are suggested with some values. The value of the EWMA plotting statistic ${F}_{t}$ is determined from the proposed continuous function $g\left({\stackrel{\smile}{\delta }}_{t}\right)$ such that the AEWMA-I CC becomes optimum in the quick recognition of the shift in the mean of the process. The working of the AEWMA-I CC is like that of the AEWMA CC proposed by^22^.

### Decision rule

In a one-sided AEWMA-I chart, if the plotting statistic $\left|{F}_{t}\right|$** >** *L,* then the process prompted the out-of-control signal. Similarly, the process prompted the out-of-control signal if the proposed statistic ${F}_{t}$ > *L* or ${F}_{t}$ **< -***L* in the case of a two-sided AEWMA-I chart.

For a specified *n* and $\phi$, the value of *L* (*L* > 0) is a threshold. The value of *L* is determined such that the in-control ARL optimal sensitivity of the AEWMA-I CC statistic $\left|{F}_{t}\right|$ is ensured at some chosen fixed level (say ARL_0_). For all specified parametric combinations of *n* and $\phi$ the value of *L* is determined separately. The parametric combinations of $\phi$, *n* with some given specified $\delta$ overwhelmingly affect the optimum in-control RL threshold performance. The *L* value is utilized to fix the in-control ARL as the ARL_0_; threshold by selecting the recommended adaptive functional methodology.

The out-of-control ARL is impacted by the value of Weibull distribution parameters, sample size, and design parameters $\phi$**,** *L***.** The trouble in concentrating out-of-control ARL is that there are two parameters in the Weibull distribution. We consider the shift level of the shape parameter ${\theta }_{1}/{\theta }_{0}$ = 1. This is sensible since, in applied applications, the scale parameter will undoubtedly change as a result of assignable causes, while the shape parameter is more related to the regular properties of the system and is somewhat stable. When keeping the shape $\theta$ as a constant, the value of out-of-control ARL of the AEWMA-I chart with transformed Weibull data relies upon the shift level of the scale parameter ${\eta }_{1}/{\eta }_{0}.$

### Performance evaluation

The RL profile are suitable measures to evaluate the performance of a CC. The Probability method, Integral equations method, Markov Chain method, and the Monte Carlo (MC) simulations method are common methods in SPC literature. In this manuscript, we use the MC method to calculate the ARL, and SDRL of the EWMA and AEWMA-I CC as being a broadly used method. The in-control ARL (${ARL}_{0}$) is set to 370 with sample sizes *n* = 3, 4, 5. We have taken the values of $\phi =$ 0.15 and 0.20 as the function performs well for $\phi$ less than or equal to 0.2. In this paper, we have taken different shifts in scales denoted with (${\eta }_{1}/{\eta }_{0}$) and shifts in parameters i.e. $\theta .$ Table 1 presents the RL profile of the EWMA CC with $\phi =0.15\mathrm{ and} n={3,4},5.$ Appendix Table 1 presented the ARLs and SDRLs of EWMA CC with $\phi =0.20\mathrm{ and} n={3,4},5.$ For the proposed AEWMA-I CC, random sampling is done from two-parameter Weibull distribution with specified distribution parameters. We have fixed $\delta =$ 1.1(0.1)2.0 and $2.5 \left(0.5\right) 4.0$ with 50,000 iterations of MC simulations.

Appendix Tables 2 and 3 contain results of run length profile with MC method for the AEWMA-I CC with $\phi =$ 0.15 and 0.20 when $\delta$ of different extents entered in the process mean. Moreover, to portray the general lead of the results a short conversation is given by

- It can be noticed, from Appendix Tables 2 and 3, that the value of $\phi$ affects on the RL profiles in both in-control and out-of-control states.
- For fixed $\phi$ $,$ by increasing $\delta$, the RL profiles tend to decrease. For instance, from Appendix Table 2 with $\delta$ = 1.1 at $\phi$= 0.15 for *n* = 3, 4, 5 the respective ARL = 210.27, 188.55, 170.51, and SDRL = 198.54, 177.23, 156.87 similarly with $\delta$ = 1.2 for *n* = 3, 4, 5 the respective ARL = 112.88, 96.89, 85.24 and SDRL = 97.21, 82.16, 71.54 at ARL_0_ = 370.
- For fixed $\delta$ $,$ by increasing the value of $\phi$, the ARL is increasing and SDRL is decreasing. For instance, from Appendix Table 1 with fixed $\delta$ = 1.1 at $\phi$= 0.15 for *n* = 3, 4, 5 the respective ARL = 210.27, 188.55, 170.51 and SDRL = 198.54, 177.23, 156.87 and from Appendix Table 1 with fixed $\delta$ = 1.1 at $\phi$= 0.20 for *n* = 3, 4, 5 the ARL = 214.86, 193.81, 176.07 and SDRL = 193.37, 169.15, 152.21 at ARL_0_ = 370.
- The threshold (*L*) and ARL_0_ are directly proportional.

**Algorithm**: To work out the RL profiles of the AEWMA-I CC for Weibul process utilizing the MC technique is described in the steps given below,

**Step 1:** Fix the values of parameters, sample size (*n*), and a smoothing constant $(\phi )$.

**Step 2:** Generate an item (or subgroup of the desired size) using simple random sampling at time t and measure its quality characteristic ${U}_{t}$ using (1) with specified Weibull distribution parameters.

**Step 3:** Normalize the Weibull-distributed data using Hastings’s approximation given in (2), (3) and calculate ${V}_{t}=\sqrt{n}{\overline{Z} }_{t}$ .

**Step 4:** Estimate mean shift $$\delta$$(say $${\stackrel{\smile}{\delta }}_{t}$$) by using $${\widehat{\delta }}_{t}^{**}$$ and by using this estimated $${\stackrel{\smile}{\delta }}_{t}$$, the proposed continuous function $$g\left({\stackrel{\smile}{\delta }}_{t}\right)$$ will get the value of the smoothing constant for the AEWMA-I CC and then calculate the plotting statistic $${F}_{t}$$ ("Design of the proposed AEWMA CC for Weibull distribution" contains the details of the AEWMA-I CC design).

**For in-control process**

i. Decide the in-control ARL_0_ (say 370).
ii. Select the value of *L* (threshold). This is done so that we get a fixed level of ARL_0_ reaches some chosen fixed level (say 370). We have considered the 50,000 replicates.
iii. A similar practice is done with different parametric combinations, at some given specified $\delta ,$ before the use of the chart at phase II.

**For out-of-control process**

i. If $\left|{F}_{t}\right|>L,$ named as out-of-control, becomes RL; note the iteration number as RL. Else, recurrence steps 2–4.
ii. Continue choosing the random sampling units from MC simulation till the accomplishment of 50,000 replications.
iii. The desired average and standard deviation of RLs are computed.
iv. The RL profiles at several quantified given $\delta$ are evaluated through respective simulations.

Hence, the RL profiles stated in Appendix Tables 2 and 3 have been widely produced for the performance assessment of this study.

## Performance comparison

Performance assessment of a CC employing its RL abilities, ARL, and SDRL is quite common in SPC. On the same lines, we use RL abilities as a demonstration standard in this study. For a given ARL_0_, smoothing constant $\phi$ and the magnitude of the shift (δ), any CC is supposed to be good enough than its competitor CCs if its out-of-control ARLs are significantly lesser. The sensitivity of the CC increases as the value of the EWMA parameter $\phi$ decreases. In practice, $\phi$ is set within the interval $\left[ {0.05 \le \phi \le 0.25} \right]$ with $\phi = 0.15,$ and 0.20 being popular choices. A rule of thumb is to use the small values $\phi$ of to detect smaller shifts. (Montgomery, 2009).

The AEWMA-I CC has been evaluated with EWMA CC. The ARL_0_ = 370 with sample size *n* = 3, 4, 5. The $\phi =$ 0.15, 0.20 are taken. The 50,000 iterations are done to calculate the RL attributes of CCs..

The revelations connected with the outcomes are examined under:

i. From Table 2, when ARL_0_ = 370 and $\phi =$ 0.15, the increase in sample size from 3 to 5 and increase in shape parameter from 0.5 to 3 resulted in a significant decrease in ARL and SDRL of AEWMA-I as compared to EWMA on all scale shifts from 1.1 to 4.5. For instance, with $\theta =0.5, {\eta }_{1}/{\eta }_{0}=1.1$, the ARLs are 247.77 and 175.63 while at shift 4.5 the ARLs are 4.58 and 3.54 for EWMA and AEWMA-I CCs respectively.
ii. Similarly, same result has been observed in Appendix Table 4, when ARL_0_ = 370 and $\phi =$ 0.20.

It very well may be seen from Appendix Tables 3 and 4 that the AEWMA-I chart performs uniformly and extensively better than the EWMA CC in all cases, which shows the predominance of the AEWMA-I chart over its counterpart.

## Illustrative example

In this section, a typical practice that has been trailed by various researchers to clarify the execution and working of the CCs with the assistance of real datasets. To clarify the operation and implementation of the suggested CC, we take a look at a real-world dataset. Observing the breaking strength of the fibrous composite is vital in the ventures that ensure the security of material utilized in the aero industry and construction of bridges.

For this purpose, the real-life dataset used by^26, 27^ is considered. It is a dataset related to carbon fibers' breaking strengths in manufacturing fibrous composite materials. This is obtained from an investigation by the U.S. Armed Force Materials Technology Laboratory in Watertown, Massachusetts. We assume the data set incorporates 20 samples with a sample size *n* = 5 is the Weibull process with scale parameter ($\eta$ = 2.9437) and shape parameter ($\theta$ = 2.7929). The Anderson–Darling test is applied to check the goodness of fit that results in the p-value of 0.8306, so the data fits well to the Weibull distribution. Draw 15 random-size samples (n = 5) without replacement and consider them in-control samples. Now contaminate the data by adding 1 in each observation, draw 10 random samples of size n = 5 without replacement, and consider them as out-of-control samples. The parameter estimation is done by the maximum likelihood estimation (MLE) method.

**Figure 1:**
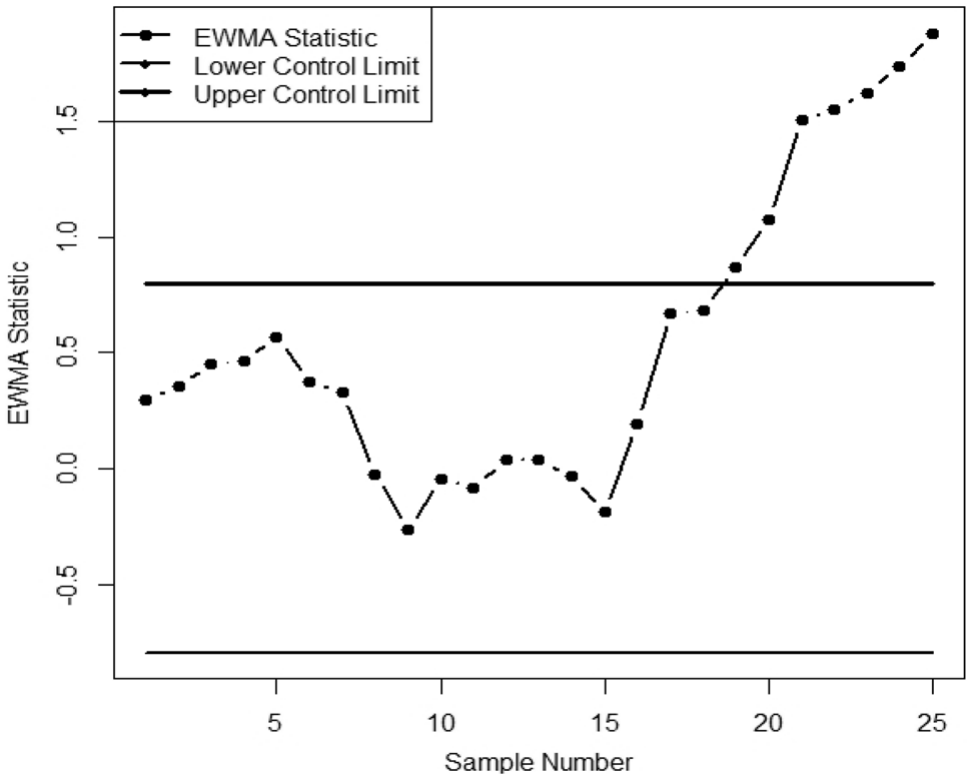
AEWMA-I chart for given data.

**Figure 2:**
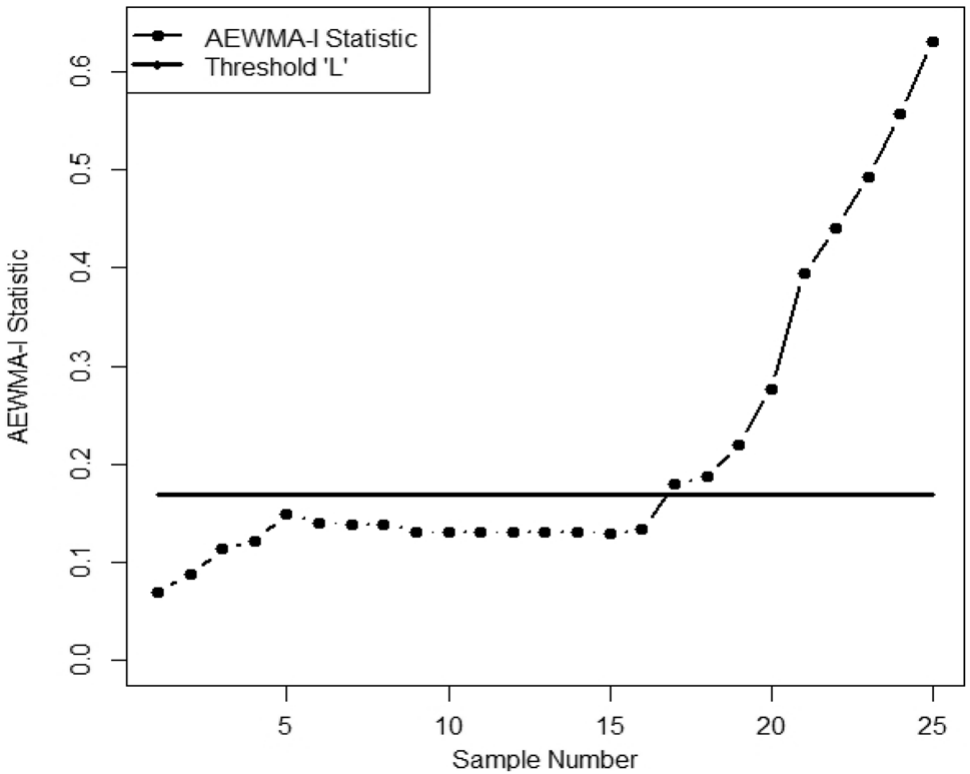
EWMA chart for given data.

We apply two competing CCs on this data with an ARL_0_ = 370. The parameter choices considered for the CCs are EWMA with ($\phi$= 0.15, *LCL* = -0.7971, *UCL* = 0.7971); and the AEWMA-I with ($\phi$ = 0.15, *L* = 0.1685). The resultant values CCs are given in Appendix Table 6 and demonstrated in Figs. 1 and 2 following the design given in "Design of the proposed control chart for Weibull distribution". 2. It is evident from Figures 1 and 2 that during the first 15 samples, both CCs stay constant. Nevertheless, both CCs start to show out-of-control signals in the process mean after that point. Interestingly, the AEWMA-I chart exhibits a faster out-of-control signal than its corresponding chart. An out-of-control signal is triggered at the 17th and 19th samples by the AEWMA-I chart, respectively.

## Main findings

The extensive simulation results are obtained to evaluate the performance of the proposal,the results of the proposed chart are discussed as:

i. i. It is qualified to rapidly identify small shifts to monitor mean in the process in which the under-study variable follows the Weibull distribution. For instance, from Table 2, at scale shift, 10%, *n* = 5, $\theta =$ 0.5, the ARL of the AEWMA-I chart is 175.63 with $\phi =$ 0.20, and at similar parameters, the ARL of EWMA is 247.57, which legitimizes the qualification of speedy detection of small shift for monitoring of the process mean.
ii. Appendix Tables 2 and 3 show that as the shift in the mean is increased, the ARL values are decreased, showing that process shift is indicated at an early stage with a larger change in mean. For example, from Appendix Table 2 at scale shift 10%, *n* = 5, and $\theta =$ 0.5, the ARL is 175.63 with $\phi =$ 0.20, and at scale shift 400% with similar parameters, the ARL is 3.50. Therefore, this chart performance is equal for small and large shifts in the mean.
iii. The width of control limits of AEWMA-I is more modest than those of EWMA. Like, from the illustrative example control limit width of the AEWMA-I chart is 0.3370 while the quite far width of EWMA is 1.5942, which shows that this chart is more delicate, having thin control limits.
iv. From Appendix Tables 2 and 3, the ARL decline with the increase in sample size (*n*). For instance, from Appendix Table 2, at scale shift 10%,$\theta =$ 1.0 and $\phi =$ 0.15, the ARL are (107.26, 91.91, 81.00) at sample size (*n* = 3, 4, 5). The ARL increases as the smoothing constant $(\phi )$ increases. For instance, from Appendix Tables 2 and 3, at scale shift 10%,$\theta =$ 1.0 and *n* = 3 the ARL are (107.26, 111.02) at smoothing constant ($\phi =$ 0.15, 0.20).
v. It is seen that, for a fixed value of sample size and smoothing constant, ARL profiles are less than its competitor on all scale shifts. For instance, from Appendix Table 3 at scale shift 10%, for *n* = 5,$\phi =$ 0.15, and $\theta =$ 1.0, the ARL of the AEWMA-I chart is 81.00, and at the similar parameters in Appendix Table 3, the ARL of EWMA is 130.24. This demonstrates the proficiency of this chart over the past one.
vi. It is noticed that, for a fixed value of sample size and shape parameter, ARL profiles are good than their competitor on all scale shifts.. For instance, from Appendix Table 4 at scale shift 40%, for *n* = 5,$\theta =$ 2.0, the ARL= 33.24 for proposed CC with $\phi =$ 0.20. At the similar parameters in Appendix Table 4, the ARL of EWMA is 47.69. This demonstrates the proficiency of this chart over the past one.
vii. For smoothing constant and shape parameter, ARL profiles are less than its competitor on all scale shifts. For instance, from Appendix Table 3 at scale shift 80%, for *n* = 4,$\phi =$ 0.15, and $\theta =$ 1.0 the ARL of the AEWMA-I chart is 91.91 and at the similar parameters in Appendix Table 3, the ARL of EWMA is 146.80. This demonstrates the proficiency of this chart over the past one. Similar results are observed in Appendix Table 5 for the expected values of the ARLs and SDRLs.

## Conclusion

The adaptive CCs have procured great thought as they are not simply more fragile than the non-adaptive CCs. They are valuable in providing better security when the shift in the process is dependent upon existing in some range. In the current work, we have suggested the AEWMA-I CC to monitor irregular variations in the process's mean, which follows the Weibull distribution. First, the data ought to be normalized utilizing Hasting transformation. The MC simulation method is used for RL profile calculation. During the fair assessment of the RL profiles, it was observed that the AEWMA-I CC with transformed Weibull data performs better than the EWMA chart in recognizing the changes in scale parameter when the shape parameter is fixed. The numerical example based on industrial data is given to delineate using the AEWMA-I CC. Based on the current research, AEWMA charts that simultaneously observe the mean and variance or screen shift in the process variance can be created. It may be possible to extend the current work to create new AEWMA charts for additional processes that are not generally/normally distributed. Additionally, new AEWMA charts for additional non-normal bivariate and multivariate distributions could be planned using the current work as a basis. In the current study, we presented AEWMA-I CC for monitoring the process means which follow the Weibull distribution. For further study, we can monitor the process dispersion CCs such as variance and Coefficient-of-variation CCs by considering non-normal processes.

### Supplementary Information

Supplementary Tables.

## Data Availability

The datasets used and/or analyzed during the current study are available from the corresponding author upon reasonable request. Further, no experiments on humans and/or the use of human tissue samples were involved in this study.
