# Supplementary material for: A Weibull process monitoring with AEWMA control chart: an application to breaking strength of the fibrous composite
Source: Sci Rep. 2023 Nov 14;13:19873. doi: 10.1038/s41598-023-47159-9 (PMC10645773; doi:10.1038/s41598-023-47159-9)
Supplement: Supplementary file 1 — Supplementary Tables. [file 41598_2023_47159_MOESM1_ESM.docx]

**Appendix:**

| **Table 1.** The RL profile of the EWMA chart for $\phi$ = 0.20, *L* = 2.8589, and subgroup size *n* = 3, 4, 5. | | | | | | | | | |
| --- | --- | --- | --- | --- | --- | --- | --- | --- | --- |
| **Scale Shift (**${\boldsymbol{\eta}_{\boldsymbol{1}}}/{\boldsymbol{\eta}_{\boldsymbol{0}}}$**)** | ***n*** | **Shape Parameter =** $\boldsymbol{\theta}$ | | | | | | | |
|  |  | **0.5** | **1** | **1.5** | **2** | **2.5** | **3** | **3.5** | **4** |
|  |  | **ARL** | **ARL** | **ARL** | **ARL** | **ARL** | **ARL** | **ARL** | **ARL** |
|  |  | **(SDRL)** | **(SDRL)** | **(SDRL)** | **(SDRL)** | **(SDRL)** | **(SDRL)** | **(SDRL)** | **(SDRL)** |
| 1 | 3 | 368.60 (366.02) | 370.66 (364.54) | 371.71 (368.17) | 370.22 (368.66) | 369.22 (365.44) | 370.10 (366.22) | 372.85 (368.10) | 369.57 (364.48) |
|  | 4 | 368.37 (361.66) | 369.63 (365.29) | 368.12 (363.09) | 368.56 (362.07) | 370.71 (365.12) | 369.71 (365.23) | 369.76 (365.99) | 369.90 (366.17) |
|  | 5 | 369.49 (362.72) | 369.82 (362.24) | 370.89 (366.63) | 368.21 (364.00) | 369.21 (364.96) | 368.98 (364.46) | 369.42 (362.92) | 368.65 (363.16) |
| 1.1 | 3 | 283.11 (279.50) | 180.80 (177.51) | 111.22 (106.23) | 71.28 (66.45) | 47.96 (42.98) | 34.12 (29.45) | 25.33 (20.89) | 19.62 (15.27) |
|  | 4 | 270.11 (263.12) | 158.34 (153.84) | 92.94 (88.22) | 57.19 (52.12) | 38.01 (33.15) | 26.80 (22.23) | 19.98 (15.66) | 15.63 (11.51) |
|  | 5 | 258.18 (254.98) | 143.28 (138.06) | 79.71 (74.89) | 47.69 (42.50) | 31.55 (26.78) | 22.20 (17.87) | 16.41 (12.24) | 12.91 (9.07) |
| 1.2 | 3 | 187.52 (182.67) | 76.34 (71.19) | 36.81 (32.10) | 21.34 (16.97) | 14.14 (10.29) | 10.21 (6.80) | 7.83 (4.82) | 6.33 (3.61) |
|  | 4 | 166.16 (161.24) | 61.54 (56.43) | 29.03 (24.32) | 16.86 (12.77) | 11.29 (7.68) | 8.25 (5.09) | 6.47 (3.64) | 5.23 (2.75) |
|  | 5 | 150.23 (144.11) | 51.73 (46.81) | 23.94 (19.30) | 14.09 (10.10) | 9.47 (6.02) | 7.00 (4.00) | 5.55 (2.90) | 4.56 (2.22) |
| 1.4 | 3 | 86.92 (82.36) | 25.01 (20.53) | 11.76 (8.21) | 7.22 (4.30) | 5.13 (2.72) | 3.95 (1.88) | 3.18 (1.42) | 2.68 (1.13) |
|  | 4 | 70.96 (66.08) | 19.62 (15.39) | 9.43 (6.07) | 5.93 (3.23) | 4.27 (2.04) | 3.33 (1.45) | 2.74 (1.12) | 2.32 (0.91) |
|  | 5 | 60.19 (55.29) | 16.28 (12.17) | 7.96 (4.80) | 5.10 (2.55) | 3.74 (1.68) | 2.94 (1.20) | 2.43 (0.93) | 2.07 (0.77) |
| 1.8 | 3 | 32.28 (27.85) | 9.02 (5.81) | 4.79 (2.47) | 3.21 (1.44) | 2.40 (1.00) | 1.91 (0.77) | 1.59 (0.64) | 1.37 (0.53) |
|  | 4 | 25.39 (20.96) | 7.31 (4.29) | 4.01 (1.89) | 2.73 (1.12) | 2.08 (0.80) | 1.68 (0.65) | 1.40 (0.53) | 1.22 (0.42) |
|  | 5 | 20.96 (16.61) | 6.25 (3.46) | 3.50 (1.53) | 2.44 (0.93) | 1.87 (0.69) | 1.51 (0.57) | 1.27 (0.46) | 1.12 (0.33) |
| 2.5 | 3 | 13.97 (10.13) | 4.50 (2.26) | 2.62 (1.10) | 1.83 (0.74) | 1.41 (0.55) | 1.18 (0.39) | 1.07 (0.26) | 1.03 (0.16) |
|  | 4 | 11.12 (7.48) | 3.79 (1.75) | 2.27 (0.88) | 1.60 (0.62) | 1.25 (0.45) | 1.08 (0.28) | 1.02 (0.15) | 1.01 (0.07) |
|  | 5 | 9.36 (5.93) | 3.34 (1.43) | 2.02 (0.75) | 1.44 (0.55) | 1.15 (0.36) | 1.04 (0.19) | 1.01 (0.08) | 1.00 (0.04) |
| 4.5 | 3 | 6.04 (3.39) | 2.33 (0.96) | 1.43 (0.57) | 1.11 (0.31) | 1.02 (0.15) | 1.00 (0.07) | 1.00 (0.03) | 1.00 (0.01) |
|  | 4 | 5.00 (2.55) | 2.02 (0.78) | 1.27 (0.46) | 1.04 (0.20) | 1.00 (0.07) | 1.00 (0.02) | 1.00 (0.01) | 1.00 (0.00) |
|  | 5 | 4.35 (2.08) | 1.82 (0.67) | 1.16 (0.37) | 1.01 (0.12) | 1.00 (0.03) | 1.00 (0.01) | 1.00 (0.00) | 1.00 (0.00) |

| **Table 2.** The RL profile of the AEWMA-I chart for $\phi$ = 0.15, *L* = 0.1685, and subgroup size n = 3, 4, 5. | | | | | | | | | |
| --- | --- | --- | --- | --- | --- | --- | --- | --- | --- |
| **Scale Shift (**${\boldsymbol{\eta}_{\boldsymbol{1}}}/{\boldsymbol{\eta}_{\boldsymbol{0}}}$**)** | ***n*** | **Shape Parameter =** $\boldsymbol{\theta}$ | | | | | | | |
|  |  | **0.5** | **1** | **1.5** | **2** | **2.5** | **3** | **3.5** | **4** |
|  |  | **ARL** | **ARL** | **ARL** | **ARL** | **ARL** | **ARL** | **ARL** | **ARL** |
|  |  | **(SDRL)** | **(SDRL)** | **(SDRL)** | **(SDRL)** | **(SDRL)** | **(SDRL)** | **(SDRL)** | **(SDRL)** |
| 1 | 3 | 369.63 (354.54) | 369.91 (355.55) | 370.51 (356.08) | 370.85 (359.67) | 370.10 (358.30) | 370.44 (357.25) | 369.71 (354.82) | 369.81 (360.51) |
|  | 4 | 369.96 (358.38) | 369.46 (359.49) | 370.33 (355.68) | 369.12 (357.51) | 369.34 (355.67) | 369.58 (354.26) | 369.85 (357.03) | 370.34 (360.29) |
|  | 5 | 369.66 (355.94) | 369.08 (356.64) | 369.67 (357.34) | 370.36 (356.81) | 369.48 (357.17) | 369.65 (357.25) | 369.32 (355.83) | 369.63 (355.88) |
| 1.1 | 3 | 211.47 (186.52) | 110.23 (86.41) | 68.47 (50.41) | 46.82 (33.59) | 34.04 (24.45) | 25.64 (18.40) | 20.00 (14.28) | 15.98 (11.55) |
|  | 4 | 190.74 (165.69) | 94.96 (72.00) | 57.49 (41.60) | 38.48 (27.41) | 27.70 (19.75) | 20.83 (14.92) | 16.08 (11.53) | 12.91 (9.15) |
|  | 5 | 175.63 (148.20) | 84.07 (61.93) | 49.88 (35.30) | 33.17 (23.44) | 23.70 (16.75) | 17.52 (12.48) | 13.59 (9.58) | 10.86 (7.57) |
| 1.2 | 3 | 116.27 (91.46) | 49.48 (35.63) | 27.32 (19.78) | 17.29 (12.54) | 11.89 (8.53) | 8.64 (6.07) | 6.65 (4.54) | 5.35 (3.55) |
|  | 4 | 99.87 (76.36) | 41.38 (29.26) | 22.32 (16.01) | 13.82 (9.82) | 9.48 (6.61) | 7.01 (4.70) | 5.43 (3.51) | 4.36 (2.77) |
|  | 5 | 88.57 (66.03) | 35.37 (25.08) | 18.97 (13.34) | 11.76 (8.20) | 8.02 (5.43) | 5.88 (3.81) | 4.60 (2.86) | 3.72 (2.28) |
| 1.3 | 3 | 75.70 (56.29) | 29.28 (20.92) | 15.20 (11.04) | 9.30 (6.56) | 6.40 (4.35) | 4.72 (3.09) | 3.68 (2.34) | 2.99 (1.89) |
|  | 4 | 64.18 (46.45) | 23.94 (17.05) | 12.20 (8.59) | 7.47 (5.11) | 5.18 (3.34) | 3.85 (2.40) | 3.03 (1.86) | 2.43 (1.49) |
|  | 5 | 55.96 (40.17) | 20.27 (14.29) | 10.26 (7.09) | 6.34 (4.12) | 4.39 (2.73) | 3.30 (2.02) | 2.58 (1.56) | 2.11 (1.26) |
| 1.4 | 3 | 55.23 (39.91) | 19.76 (14.28) | 10.01 (7.12) | 6.14 (4.13) | 4.21 (2.74) | 3.18 (2.01) | 2.47 (1.55) | 2.02 (1.24) |
|  | 4 | 46.18 (32.95) | 15.91 (11.36) | 8.00 (5.47) | 4.97 (3.19) | 3.46 (2.16) | 2.61 (1.60) | 2.05 (1.24) | 1.68 (0.97) |
|  | 5 | 40.00 (28.26) | 13.38 (9.45) | 6.77 (4.48) | 4.22 (2.61) | 2.97 (1.79) | 2.23 (1.34) | 1.77 (1.03) | 1.47 (0.78) |
| 1.5 | 3 | 42.78 (30.79) | 14.49 (10.45) | 7.29 (5.00) | 4.48 (2.90) | 3.15 (1.98) | 2.36 (1.47) | 1.88 (1.13) | 1.56 (0.88) |
|  | 4 | 35.26 (25.04) | 11.63 (8.25) | 5.86 (3.83) | 3.67 (2.28) | 2.58 (1.58) | 1.95 (1.17) | 1.57 (0.88) | 1.33 (0.65) |
|  | 5 | 30.15 (21.30) | 9.76 (6.76) | 4.97 (3.13) | 3.13 (1.89) | 2.22 (1.34) | 1.69 (0.96) | 1.38 (0.69) | 1.20 (0.49) |
| 1.6 | 3 | 34.48 (24.71) | 11.33 (8.07) | 5.66 (3.79) | 3.55 (2.25) | 2.50 (1.56) | 1.89 (1.14) | 1.52 (0.85) | 1.31 (0.63) |
|  | 4 | 28.22 (20.09) | 9.02 (6.24) | 4.61 (2.91) | 2.91 (1.79) | 2.06 (1.24) | 1.58 (0.89) | 1.31 (0.63) | 1.16 (0.43) |
|  | 5 | 24.14 (17.18) | 7.61 (5.12) | 3.91 (2.40) | 2.50 (1.50) | 1.77 (1.03) | 1.39 (0.70) | 1.19 (0.47) | 1.09 (0.31) |
| 1.7 | 3 | 28.86 (20.81) | 9.18 (6.46) | 4.66 (3.06) | 2.92 (1.83) | 2.06 (1.27) | 1.59 (0.91) | 1.33 (0.65) | 1.17 (0.45) |
|  | 4 | 23.41 (16.73) | 7.37 (4.99) | 3.78 (2.35) | 2.41 (1.49) | 1.72 (1.00) | 1.36 (0.68) | 1.17 (0.45) | 1.08 (0.30) |
|  | 5 | 19.84 (14.06) | 6.23 (4.09) | 3.22 (1.95) | 2.06 (1.23) | 1.50 (0.81) | 1.22 (0.52) | 1.09 (0.32) | 1.04 (0.20) |
| 1.8 | 3 | 24.52 (17.63) | 7.65 (5.30) | 3.93 (2.52) | 2.49 (1.57) | 1.78 (1.07) | 1.40 (0.73) | 1.20 (0.49) | 1.10 (0.33) |
|  | 4 | 19.96 (14.23) | 6.23 (4.11) | 3.23 (1.98) | 2.06 (1.24) | 1.50 (0.81) | 1.23 (0.52) | 1.10 (0.33) | 1.04 (0.21) |
|  | 5 | 16.87 (11.94) | 5.27 (3.37) | 2.76 (1.66) | 1.77 (1.03) | 1.33 (0.64) | 1.12 (0.38) | 1.05 (0.22) | 1.02 (0.13) |
| 1.9 | 3 | 21.34 (15.39) | 6.63 (4.51) | 3.41 (2.18) | 2.17 (1.34) | 1.57 (0.89) | 1.28 (0.59) | 1.13 (0.39) | 1.06 (0.25) |
|  | 4 | 17.21 (12.27) | 5.35 (3.48) | 2.80 (1.73) | 1.80 (1.07) | 1.34 (0.66) | 1.14 (0.41) | 1.05 (0.24) | 1.02 (0.14) |
|  | 5 | 14.55 (10.28) | 4.58 (2.86) | 2.40 (1.44) | 1.57 (0.87) | 1.21 (0.50) | 1.07 (0.28) | 1.02 (0.15) | 1.01 (0.08) |
| 2 | 3 | 18.98 (13.69) | 5.83 (3.89) | 3.03 (1.91) | 1.93 (1.18) | 1.43 (0.75) | 1.19 (0.48) | 1.08 (0.30) | 1.03 (0.19) |
|  | 4 | 15.19 (10.81) | 4.73 (3.01) | 2.48 (1.52) | 1.62 (0.92) | 1.24 (0.54) | 1.09 (0.32) | 1.03 (0.18) | 1.01 (0.10) |
|  | 5 | 12.80 (8.97) | 4.03 (2.48) | 2.14 (1.28) | 1.42 (0.73) | 1.14 (0.40) | 1.04 (0.22) | 1.01 (0.11) | 1.00 (0.06) |
| 2.5 | 3 | 11.73 (8.37) | 3.70 (2.36) | 1.95 (1.19) | 1.34 (0.66) | 1.12 (0.36) | 1.04 (0.20) | 1.01 (0.11) | 1.00 (0.06) |
|  | 4 | 9.40 (6.60) | 3.03 (1.86) | 1.64 (0.93) | 1.18 (0.47) | 1.05 (0.23) | 1.01 (0.11) | 1.00 (0.05) | 1.00 (0.03) |
|  | 5 | 7.95 (5.34) | 2.60 (1.56) | 1.43 (0.75) | 1.10 (0.34) | 1.02 (0.15) | 1.00 (0.06) | 1.00 (0.02) | 1.00 (0.00) |
| 3 | 3 | 8.69 (6.10) | 2.78 (1.75) | 1.52 (0.84) | 1.14 (0.41) | 1.04 (0.20) | 1.01 (0.10) | 1.00 (0.05) | 1.00 (0.02) |
|  | 4 | 6.89 (4.64) | 2.29 (1.40) | 1.31 (0.62) | 1.06 (0.26) | 1.01 (0.11) | 1.00 (0.04) | 1.00 (0.01) | 1.00 (0.01) |
|  | 5 | 5.85 (3.78) | 1.97 (1.17) | 1.18 (0.47) | 1.03 (0.17) | 1.00 (0.06) | 1.00 (0.01) | 1.00 (0.01) | 1.00 (0.00) |
| 3.5 | 3 | 6.92 (4.72) | 2.25 (1.40) | 1.31 (0.63) | 1.07 (0.27) | 1.01 (0.11) | 1.00 (0.05) | 1.00 (0.02) | 1.00 (0.01) |
|  | 4 | 5.57 (3.61) | 1.86 (1.10) | 1.16 (0.44) | 1.02 (0.16) | 1.00 (0.06) | 1.00 (0.02) | 1.00 (0.01) | 1.00 (0.00) |
|  | 5 | 4.73 (2.94) | 1.62 (0.91) | 1.09 (0.31) | 1.01 (0.09) | 1.00 (0.03) | 1.00 (0.01) | 1.00 (0.00) | 1.00 (0.00) |
| 4 | 3 | 5.82 (3.88) | 1.94 (1.18) | 1.19 (0.48) | 1.03 (0.19) | 1.01 (0.07) | 1.00 (0.03) | 1.00 (0.01) | 1.00 (0.00) |
|  | 4 | 4.74 (3.01) | 1.62 (0.92) | 1.09 (0.31) | 1.01 (0.10) | 1.00 (0.03) | 1.00 (0.01) | 1.00 (0.00) | 1.00 (0.00) |
|  | 5 | 4.02 (2.46) | 1.42 (0.73) | 1.04 (0.22) | 1.00 (0.05) | 1.00 (0.01) | 1.00 (0.00) | 1.00 (0.00) | 1.00 (0.00) |

**Table 3.** The RL profile of the AEWMA-I chart for $\phi$ = 0.20, *L* = 0.2039, and subgroup size n = 3, 4, 5

| **Scale Shift (**${\boldsymbol{\eta}_{\boldsymbol{1}}}/{\boldsymbol{\eta}_{\boldsymbol{0}}}$**)** | ***n*** | **Shape Parameter =** $\boldsymbol{\theta}$ | | | | | | | |
| --- | --- | --- | --- | --- | --- | --- | --- | --- | --- |
|  |  | **0.5** | **1** | **1.5** | **2** | **2.5** | **3** | **3.5** | **4** |
|  |  | **ARL** | **ARL** | **ARL** | **ARL** | **ARL** | **ARL** | **ARL** | **ARL** |
|  |  | **(SDRL)** | **(SDRL)** | **(SDRL)** | **(SDRL)** | **(SDRL)** | **(SDRL)** | **(SDRL)** | **(SDRL)** |
| 1 | 3 | 369.15 (341.99) | 369.29 (347.59) | 369.71 (347.41) | 369.45 (343.48) | 370.63 (344.29) | 369.75 (344.14) | 370.74 (344.74) | 369.64 (344.16) |
|  | 4 | 369.64 (345.63) | 369.45 (342.35) | 369.57 (344.93) | 369.12 (340.78) | 370.58 (343.26) | 370.83 (347.13) | 369.88 (347.18) | 369.17 (344.59) |
|  | 5 | 369.84 (345.32) | 369.58 (342.36) | 369.75 (345.65) | 369.14 (342.20) | 369.50 (346.38) | 370.15 (344.70) | 369.83 (344.97) | 369.33 (344.90) |
| 1.1 | 3 | 216.49 (184.55) | 113.01 (84.62) | 70.55 (48.57) | 48.99 (32.56) | 36.07 (23.45) | 27.75 (17.97) | 21.92 (14.24) | 17.78 (11.48) |
|  | 4 | 196.15 (162.79) | 97.84 (70.57) | 59.65 (39.62) | 40.73 (26.46) | 29.89 (19.16) | 22.71 (14.60) | 17.81 (11.38) | 14.43 (9.23) |
|  | 5 | 178.59 (146.62) | 86.43 (60.80) | 52.33 (34.220 | 35.48 (22.70) | 25.65 (16.40) | 19.45 (12.32) | 15.10 (9.57) | 12.21 (7.67) |
| 1.2 | 3 | 119.39 (89.53) | 51.94 (34.66) | 29.57 (19.07) | 19.03 (12.28) | 13.26 (8.57) | 9.84 (6.25) | 7.58 (4.76) | 6.10 (3.84) |
|  | 4 | 102.69 (74.79) | 43.44 (28.20) | 24.27 (15.56) | 15.53 (9.91) | 10.74 (6.77) | 7.99 (4.94) | 6.20 (3.76) | 4.97 (3.01) |
|  | 5 | 90.95 (64.52) | 37.65 (24.07) | 20.73 (13.11) | 13.19 (8.32) | 9.11 (5.62) | 6.77 (4.10) | 5.25 (3.14) | 4.22 (2.54) |
| 1.3 | 3 | 78.56 (55.02) | 31.20 (20.28) | 16.88 (10.94) | 10.55 (6.76) | 7.24 (4.53) | 5.39 (3.36) | 4.16 (2.62) | 3.33 (2.13) |
|  | 4 | 66.81 (45.02) | 25.82 (16.60) | 13.73 (8.70) | 8.55 (5.27) | 5.93 (3.59) | 4.37 (2.67) | 3.39 (2.11) | 2.71 (1.72) |
|  | 5 | 58.30 (38.52) | 22.09 (14.03) | 11.64 (7.24) | 7.23 (4.39) | 5.03 (3.01) | 3.71 (2.26) | 2.88 (1.79) | 2.28 (1.45) |
| 1.4 | 3 | 57.25 (38.61) | 21.59 (13.93) | 11.23 (7.18) | 7.01 (4.39) | 4.83 (3.02) | 3.54 (2.24) | 2.74 (1.78) | 2.17 (1.42) |
|  | 4 | 48.24 (31.64) | 17.59 (11.24) | 9.06 (5.66) | 5.64 (3.45) | 3.92 (2.41) | 2.87 (1.82) | 2.22 (1.42) | 1.78 (1.11) |
|  | 5 | 41.92 (27.14) | 14.96 (9.44) | 7.72 (4.68) | 4.84 (2.88) | 3.33 (2.05) | 2.44 (1.54) | 1.88 (1.18) | 1.54 (0.89) |
| 1.5 | 3 | 44.92 (29.81) | 16.03 (10.38) | 8.23 (5.20) | 5.12 (3.18) | 3.51 (2.24) | 2.58 (1.68) | 1.99 (1.28) | 1.62 (0.98) |
|  | 4 | 37.57 (24.15) | 12.98 (8.24) | 6.70 (4.08) | 4.17 (2.55) | 2.86 (1.80) | 2.10 (1.34) | 1.63 (0.98) | 1.36 (0.72) |
|  | 5 | 32.46 (20.67) | 11.10 (6.90) | 5.69 (3.41) | 3.56 (2.15) | 2.44 (1.53) | 1.79 (1.10) | 1.42 (0.78) | 1.22 (0.54) |
| 1.6 | 3 | 36.86 (23.96) | 12.61 (8.09) | 6.46 (4.02) | 4.03 (2.53) | 2.74 (1.77) | 2.03 (1.32) | 1.58 (0.95) | 1.34 (0.70) |
|  | 4 | 30.17 (19.40) | 10.23 (6.41) | 5.25 (3.19) | 3.26 (2.03) | 2.23 (1.43) | 1.66 (1.01) | 1.35 (0.70) | 1.17 (0.47) |
|  | 5 | 26.19 (16.57) | 8.69 (5.32) | 4.49 (2.68) | 2.76 (1.73) | 1.90 (1.19) | 1.44 (0.80) | 1.20 (0.52) | 1.09 (0.33) |
| 1.7 | 3 | 30.68 (19.83) | 10.33 (6.62) | 5.27 (3.30) | 3.26 (2.09) | 2.24 (1.46) | 1.66 (1.03) | 1.35 (0.71) | 1.18 (0.49) |
|  | 4 | 25.50 (16.32) | 8.36 (5.19) | 4.32 (2.63) | 2.64 (1.69) | 1.83 (1.15) | 1.40 (0.76) | 1.18 (0.49) | 1.08 (0.31) |
|  | 5 | 21.70 (13.81) | 7.11 (4.31) | 3.66 (2.22) | 2.25 (1.43) | 1.56 (0.91) | 1.24 (0.58) | 1.10 (0.34) | 1.04 (0.20) |
| 1.8 | 3 | 26.41 (17.18) | 8.75 (5.52) | 4.44 (2.79) | 2.73 (1.77) | 1.88 (1.21) | 1.44 (0.81) | 1.22 (0.54) | 1.10 (0.35) |
|  | 4 | 21.73 (13.81) | 7.08 (4.33) | 3.62 (2.24) | 2.22 (1.42) | 1.55 (0.91) | 1.24 (0.58) | 1.10 (0.35) | 1.04 (0.21) |
|  | 5 | 18.63 (11.78) | 6.04 (3.63) | 3.07 (1.89) | 1.89 (1.18) | 1.36 (0.71) | 1.14 (0.41) | 1.05 (0.23) | 1.02 (0.13) |
| 1.9 | 3 | 23.21 (15.04) | 7.57 (4.76) | 3.84 (2.42) | 2.36 (1.54) | 1.64 (1.01) | 1.29 (0.64) | 1.13 (0.41) | 1.06 (0.26) |
|  | 4 | 18.96 (12.13) | 6.12 (3.72) | 3.14 (1.97) | 1.91 (1.21) | 1.38 (0.75) | 1.15 (0.44) | 1.06 (0.25) | 1.02 (0.14) |
|  | 5 | 16.17 (10.23) | 5.23 (3.10) | 2.65 (1.66) | 1.64 (0.99) | 1.24 (0.57) | 1.08 (0.30) | 1.02 (0.16) | 1.01 (0.09) |
| 2 | 3 | 20.69 (13.40) | 6.67 (4.16) | 3.36 (2.15) | 2.07 (1.35) | 1.47 (0.85) | 1.21 (0.52) | 1.08 (0.32) | 1.04 (0.20) |
|  | 4 | 16.83 (10.74) | 5.40 (3.29) | 2.74 (1.75) | 1.70 (1.04) | 1.26 (0.60) | 1.09 (0.34) | 1.03 (0.18) | 1.01 (0.10) |
|  | 5 | 14.31 (9.03) | 4.61 (2.75) | 2.32 (1.47) | 1.47 (0.83) | 1.15 (0.43) | 1.04 (0.22) | 1.01 (0.11) | 1.00 (0.06) |
| 2.5 | 3 | 13.21 (8.56) | 4.17 (2.62) | 2.10 (1.37) | 1.37 (0.74) | 1.12 (0.38) | 1.04 (0.20) | 1.01 (0.11) | 1.00 (0.06) |
|  | 4 | 10.72 (6.73) | 3.39 (2.11) | 1.72 (1.06) | 1.20 (0.51) | 1.05 (0.23) | 1.01 (0.11) | 1.00 (0.05) | 1.00 (0.02) |
|  | 5 | 9.07 (5.58) | 2.87 (1.79) | 1.48 (0.84) | 1.11 (0.36) | 1.02 (0.15) | 1.00 (0.06) | 1.00 (0.02) | 1.00 (0.01) |
| 3 | 3 | 9.74 (6.21) | 3.05 (1.97) | 1.59 (0.96) | 1.15 (0.44) | 1.04 (0.20) | 1.01 (0.09) | 1.00 (0.05) | 1.00 (0.02) |
|  | 4 | 7.92 (4.90) | 2.51 (1.60) | 1.34 (0.69) | 1.06 (0.27) | 1.01 (0.11) | 1.00 (0.04) | 1.00 (0.01) | 1.00 (0.01) |
|  | 5 | 6.72 (4.05) | 2.12 (1.35) | 1.20 (0.51) | 1.03 (0.18) | 1.00 (0.06) | 1.00 (0.02) | 1.00 (0.00) | 1.00 (0.00) |
| 3.5 | 3 | 7.87 (4.95) | 2.45 (1.60) | 1.34 (0.70) | 1.07 (0.28) | 1.01 (0.12) | 1.00 (0.05) | 1.00 (0.02) | 1.00 (0.01) |
|  | 4 | 6.39 (3.90) | 2.00 (1.27) | 1.17 (0.47) | 1.03 (0.17) | 1.00 (0.06) | 1.00 (0.02) | 1.00 (0.00) | 1.00 (0.00) |
|  | 5 | 5.41 (3.23) | 1.70 (1.04) | 1.09 (0.32) | 1.01 (0.09) | 1.00 (0.03) | 1.00 (0.01) | 1.00 (0.00) | 1.00 (0.00) |
| 4 | 3 | 6.62 (4.13) | 2.07 (1.35) | 1.20 (0.52) | 1.04 (0.20) | 1.01 (0.07) | 1.00 (0.03) | 1.00 (0.01) | 1.00 (0.00) |
|  | 4 | 5.41 (3.27) | 1.70 (1.04) | 1.10 (0.34) | 1.01 (0.11) | 1.00 (0.03) | 1.00 (0.01) | 1.00 (0.00) | 1.00 (0.00) |
|  | 5 | 4.58 (2.76) | 1.47 (0.83) | 1.05 (0.22) | 1.00 (0.06) | 1.00 (0.01) | 1.00 (0.00) | 1.00 (0.00) | 1.00 (0.00) |

**Table 4.** Relative investigation of RL profile for ARL_0_ = 370 and $\phi$ = 0.20 at different sample sizes.

| **Scale Shift (**${\boldsymbol{\eta}_{\boldsymbol{1}}}/{\boldsymbol{\eta}_{\boldsymbol{0}}}$**)** | ***n*** | **Shape Parameter =** $\boldsymbol{\theta}$ | | | | | | | |
| --- | --- | --- | --- | --- | --- | --- | --- | --- | --- |
|  |  | 0.5 | | 1 | | 2 | | 3 | |
|  |  | **EWMA** | **AEWMA-I** | **EWMA** | **AEWMA-I** | **EWMA** | **AEWMA-I** | **EWMA** | **AEWMA-I** |
|  |  | **ARL**  **(SDRL)** | **ARL**  **(SDRL)** | **ARL**  **(SDRL)** | **ARL**  **(SDRL)** | **ARL**  **(SDRL)** | **ARL**  **(SDRL)** | **ARL**  **(SDRL)** | **ARL**  **(SDRL)** |
| 1.1 | 3 | 283.11 (279.50) | 216.49 (184.55) | 180.80 (177.51) | 113.01 (84.62) | 71.28 (66.45) | 48.99 (32.56) | 34.12 (29.45) | 27.75 (17.97) |
|  | 4 | 270.11 (263.12) | 196.15 (162.79) | 158.34 (153.84) | 97.84 (70.57) | 57.19 (52.12) | 40.73 (26.46) | 26.80 (22.23) | 22.71 (14.60) |
|  | 5 | 258.18 (254.98) | 178.59 (146.62) | 143.28 (138.06) | 86.43 (60.80) | 47.69 (42.50) | 35.48 (22.70) | 22.20 (17.87) | 19.45 (12.32) |
| 1.2 | 3 | 187.52 (182.67) | 119.39 (89.53) | 76.34 (71.19) | 51.94 (34.66) | 21.34 (16.97) | 19.03 (12.28) | 10.21 (6.80) | 9.84 (6.25) |
|  | 4 | 166.16 (161.24) | 102.69 (74.79) | 61.54 (56.43) | 43.44 (28.20) | 16.86 (12.77) | 15.53 (9.91) | 8.25 (5.09) | 7.99 (4.94) |
|  | 5 | 150.23 (144.11) | 90.95 (64.52) | 51.73 (46.81) | 37.65 (24.07) | 14.09 (10.10) | 13.19 (8.32) | 7.00 (4.00) | 6.77 (4.10) |
| 1.4 | 3 | 86.92 (82.36) | 57.25 (38.61) | 25.01 (20.53) | 21.59 (13.93) | 7.22 (4.30) | 7.01 (4.39) | 3.95 (1.88) | 3.54 (2.24) |
|  | 4 | 70.96 (66.08) | 48.24 (31.64) | 19.62 (15.39) | 17.59 (11.24) | 5.93 (3.23) | 5.64 (3.45) | 3.33 (1.45) | 2.87 (1.82) |
|  | 5 | 60.19 (55.29) | 41.92 (27.14) | 16.28 (12.17) | 14.96 (9.44) | 5.10 (2.55) | 4.84 (2.88) | 2.94 (1.20) | 2.44 (1.54) |
| 1.8 | 3 | 32.28 (27.85) | 26.41 (17.18) | 9.02 (5.81) | 8.75 (5.52) | 3.21 (1.44) | 2.73 (1.77) | 1.91 (0.77) | 1.44 (0.81) |
|  | 4 | 25.39 (20.96) | 21.73 (13.81) | 7.31 (4.29) | 7.08 (4.33) | 2.73 (1.12) | 2.22 (1.42) | 1.68 (0.65) | 1.24 (0.58) |
|  | 5 | 20.96 (16.61) | 18.63 (11.78) | 6.25 (3.46) | 6.04 (3.63) | 2.44 (0.93) | 1.89 (1.18) | 1.51 (0.57) | 1.14 (0.41) |
| 2.5 | 3 | 13.97 (10.13) | 13.21 (8.56) | 4.50 (2.26) | 4.17 (2.62) | 1.83 (0.74) | 1.37 (0.74) | 1.18 (0.39) | 1.04 (0.20) |
|  | 4 | 11.12 (7.48) | 10.72 (6.73) | 3.79 (1.75) | 3.39 (2.11) | 1.60 (0.62) | 1.20 (0.51) | 1.08 (0.28) | 1.01 (0.11) |
|  | 5 | 9.36 (5.93) | 9.07 (5.58) | 3.34 (1.43) | 2.87 (1.79) | 1.44 (0.55) | 1.11 (0.36) | 1.04 (0.19) | 1.00 (0.06) |
| 4.5 | 3 | 6.04 (3.39) | 5.81 (3.62) | 2.33 (0.96) | 1.81 (1.14) | 1.11 (0.31) | 1.02 (0.14) | 1.00 (0.07) | 1.00 (0.02) |
|  | 4 | 5.00 (2.55) | 4.72 (2.87) | 2.02 (0.78) | 1.51 (0.86) | 1.04 (0.20) | 1.01 (0.07) | 1.00 (0.02) | 1.00 (0.00) |
|  | 5 | 4.35 (2.08) | 4.02 (2.41 | 1.82 (0.67) | 1.32 (0.67) | 1.01 (0.12) | 1.00 (0.03) | 1.00 (0.01) | 1.00 (0.00) |

**Table 5.** Relative investigation of Expected RL profile for ARL_0_ = 370 and $\phi$ = 0.15 at different sample sizes.

| **Scale Shift (**${\boldsymbol{\eta}_{\boldsymbol{1}}}/{\boldsymbol{\eta}_{\boldsymbol{0}}}$**)** | ***n*** | **Shape Parameter =** $\boldsymbol{\theta}$ | | | | | | | |
| --- | --- | --- | --- | --- | --- | --- | --- | --- | --- |
|  |  | 0.5 | | 1 | | 2 | | 3 | |
|  |  | **EWMA** | **AEWMA-I** | **EWMA** | **AEWMA-I** | **EWMA** | **AEWMA-I** | **EWMA** | **AEWMA-I** |
|  |  | **EARL**  **(ESDRL)** | **EARL**  **(ESDRL)** | **EARL**  **(ESDRL)** | **EARL**  **(ESDRL)** | **EARL**  **(ESDRL)** | **EARL**  **(ESDRL)** | **EARL**  **(ESDRL)** | **EARL**  **(ESDRL)** |
| 1.1 | 3 | 273.94 (267.45) | 213.12 (187.10) | 167.90 (161.23) | 110.91 (87.99) | 65.01 (58.12) | 47.00 (33.91) | 31.91 (25.92) | 26.02 (18.85) |
|  | 4 | 262.32 (254.75) | 191.24 (166.11) | 146.80 (140.73) | 95.12 (72.42) | 52.61 (46.20) | 39.51 (28.32) | 25.14 (19.25) | 20.12 (14.84) |
|  | 5 | 246.87 (240.72) | 176.21 (149.19) | 131.24 (125.89) | 84.02 (61.99) | 42.95 (36.12) | 32.29 (22.59) | 20.55 (15.31) | 17.52 (12.41) |
| 1.2 | 3 | 178.17 (169.11) | 118.19 (92.12) | 68.29 (62.16) | 49.21 (35.52) | 20.22 (15.21) | 17.21 (12.23) | 10.15 (6.15) | 8.69 (6.15) |
|  | 4 | 154.21 (149.91) | 98.12 (75.17) | 55.51 (49.47) | 41.15 (28.27) | 16.29 (11.29) | 13.59 (9.66) | 8.39 (4.77) | 7.07 (4.72) |
|  | 5 | 136.20 (130.13) | 87.17 (65.53) | 45.82 (39.99) | 34.87 (25.98) | 13.17 (8.58) | 11.68 (8.16) | 7.15 (3.72) | 5.82 (3.75) |
| 1.4 | 3 | 78.97 (72.14) | 55.13 (39.22) | 23.41 (18.12) | 19.78 (14.32) | 7.35 (4.25) | 6.23 (4.19) | 4.14 (1.82) | 3.19 (2.07) |
|  | 4 | 64.28 (57.17) | 46.37 (32.15) | 18.12 (13.22) | 15.97 (11.42) | 6.12 (3.15) | 4.75 (3.20) | 3.52 (1.44) | 2.63 (1.61) |
|  | 5 | 53.24 (46.84) | 39.24 (27.91) | 15.78 (10.95) | 13.44 (9.49) | 5.21 (2.41) | 4.27 (2.65) | 3.12 (1.19) | 2.22 (1.36) |
| 1.8 | 3 | 29.21 (23.22) | 24.31 (17.15) | 9.05 (5.25) | 7.75 (5.32) | 3.45 (1.41) | 2.42 (1.55) | 2.07 (0.77) | 1.44 (0.72) |
|  | 4 | 23.41 (18.01) | 19.55 (14.60) | 7.42 (4.07) | 6.25 (4.10) | 2.92 (1.10) | 2.01 (1.21) | 1.80 (0.64) | 1.22 (0.56) |
|  | 5 | 19.59 (14.43) | 16.59 (11.72) | 6.22 (3.15) | 5.21 (3.31) | 2.69 (0.94) | 1.75 (1.01) | 1.65 (0.58) | 1.12 (0.38) |
| 2.5 | 3 | 13.42 (9.01) | 11.53 (8.41) | 4.72 (2.25) | 3.79 (2.38) | 1.97 (0.73) | 1.32 (0.64) | 1.24 (0.44) | 1.04 (0.21) |
|  | 4 | 11.44 (6.79) | 9.48 (6.68) | 3.95 (1.72) | 3.10 (1.88) | 1.75 (0.62) | 1.18 (0.46) | 1.12 (0.32) | 1.01 (0.13) |
|  | 5 | 9.14 (5.23) | 7.28 (5.42) | 3.45 (1.41) | 2.59 (146) | 1.55 (0.56) | 1.13 (0.35) | 1.06 (0.22) | 1.00 (0.06) |
| 4.5 | 3 | 6.32 (3.19) | 5.19 (3.25) | 2.50 (0.98) | 1.75 (1.05) | 1.14 (0.35) | 1.01 (0.14) | 1.01 (0.09) | 1.00 (0.02) |
|  | 4 | 5.11 (2.23) | 4.12 (2.52) | 2.16 (0.77) | 1.46 (0.78) | 1.05 (0.23) | 1.00 (0.07) | 1.00 (0.03) | 1.00 (0.00) |
|  | 5 | 4.45 (2.01) | 3.42 (2.21) | 1.98 (0.64) | 1.29 (0.59) | 1.01 (0.15) | 1.00 (0.03) | 1.00 (0.01) | 1.00 (0.00) |

**Table 6.** Data set related to breaking strengths of carbon fibers.

| **Sample  Number** | **Breaking stresses (GPa)  of carbon fibers** | | | | | **Transformed Standard Normal  from Weibull** | | | | | $\bar{\boldsymbol{V}_{\boldsymbol{t}}}$ | $\boldsymbol{E}_{\boldsymbol{t}}$ | $\boldsymbol{F}_{\boldsymbol{t}}$ |
| --- | --- | --- | --- | --- | --- | --- | --- | --- | --- | --- | --- | --- | --- |
|  |  |  |  |  |  |  |  |  |  |  |  |  |  |
| **1** | **4.91** | **2.85** | **2.12** | **5.08** | **2.76** | **2.1603** | **0.2502** | **-0.4407** | **2.3212** | **0.1665** | **0.8915** | **0.2990** | 0.0699 |
| 2 | 3.68 | 3.15 | 1.84 | 2.97 | 2.95 | 1.0159 | 0.5277 | -0.7190 | 0.3614 | 0.3429 | 0.3058 | 0.3567 | 0.0880 |
| 3 | 3.11 | 2.95 | 4.20 | 3.19 | 1.87 | 0.4908 | 0.3429 | 1.4964 | 0.5646 | -0.6886 | 0.4412 | 0.4512 | 0.1135 |
| 4 | 3.56 | 5.56 | 1.17 | 2.93 | 1.22 | 0.9054 | 2.7800 | -1.4527 | 0.3243 | -1.3927 | 0.2329 | 0.4616 | 0.1217 |
| 5 | 3.31 | 4.38 | 3.15 | 3.33 | 1.59 | 0.6752 | 1.6636 | 0.5277 | 0.6936 | -0.9785 | 0.5163 | 0.5656 | 0.1491 |
| 6 | 2.83 | 2.55 | 3.11 | 1.73 | 1.18 | 0.2316 | -0.0297 | 0.4908 | -0.8316 | -1.4406 | -0.3159 | 0.3748 | 0.1396 |
| 7 | 2.50 | 2.79 | 2.17 | 2.48 | 3.19 | -0.0767 | 0.1944 | -0.3921 | -0.0956 | 0.5646 | 0.0389 | 0.3316 | 0.1392 |
| 8 | 2.38 | 1.08 | 1.18 | 3.19 | 0.81 | -0.1904 | -1.5638 | -1.4406 | 0.5646 | -1.9297 | -0.9120 | -0.0240 | 0.1391 |
| 9 | 2.82 | 0.39 | 1.41 | 2.43 | 2.73 | 0.2223 | -2.6946 | -1.1744 | -0.1429 | 0.1386 | -0.7302 | -0.2653 | 0.1313 |
| 10 | 2.35 | 0.81 | 3.19 | 5.08 | 4.70 | -0.2189 | -1.9297 | 0.5646 | 2.3212 | 1.9627 | 0.5400 | -0.0444 | 0.1314 |
| 11 | 1.57 | 3.39 | 2.03 | 2.48 | 2.81 | -0.9998 | 0.7489 | -0.5290 | -0.0956 | 0.2130 | -0.1325 | -0.0822 | 0.1312 |
| 12 | 2.97 | 2.03 | 2.79 | 3.15 | 3.75 | 0.3614 | -0.5290 | 0.1944 | 0.5277 | 1.0804 | 0.3270 | 0.0398 | 0.1313 |
| 13 | 3.56 | 2.00 | 1.89 | 2.76 | 2.79 | 0.9054 | -0.5587 | -0.6685 | 0.1665 | 0.1944 | 0.0078 | 0.0365 | 0.1313 |
| 14 | 3.31 | 2.48 | 3.28 | 0.39 | 3.15 | 0.6752 | -0.0956 | 0.6476 | -2.6946 | 0.5277 | -0.1879 | -0.0320 | 0.1312 |
| 15 | 3.51 | 0.81 | 2.81 | 0.85 | 2.95 | 0.8594 | -1.9297 | 0.2130 | -1.8716 | 0.3429 | -0.4772 | -0.1873 | 0.1292 |
| 16 | 2.59 | 3.03 | 3.48 | 5.42 | 4.11 | 0.0078 | 0.4169 | 0.8317 | 2.6454 | 1.4130 | 1.0630 | 0.1973 | 0.1333 |
| 17 | 4.39 | 4.33 | 3.05 | 4.68 | 4.60 | 1.6730 | 1.6171 | 0.4354 | 1.9439 | 1.8690 | 1.5077 | 0.6734 | 0.1793 |
| 182 | 3.81 | 4.09 | 2.57 | 2.25 | 1.98 | 1.1358 | 1.3945 | -0.0109 | -0.3147 | -0.5785 | 0.3252 | 0.6815 | 0.1871 |
| 19 | 3.00 | 3.38 | 2.36 | 4.22 | 4.65 | 0.3891 | 0.7397 | -0.2094 | 1.5149 | 1.9158 | 0.8700 | 0.8711 | 0.2204 |
| 20 | 3.76 | 3.88 | 3.95 | 2.89 | 3.83 | 1.0897 | 1.2003 | 1.2650 | 0.2873 | 1.1542 | 0.9993 | 1.0756 | 0.2763 |
| 21 | 4.60 | 4.39 | 4.68 | 5.20 | 3.59 | 1.8690 | 1.6730 | 1.9439 | 2.4352 | 0.9330 | 1.7708 | 1.5082 | 0.3945 |
| 22 | 2.92 | 3.59 | 2.69 | 5.20 | 2.89 | 0.3151 | 0.9330 | 0.1013 | 2.4352 | 0.2873 | 0.8144 | 1.5551 | 0.4407 |
| 23 | 4.75 | 1.98 | 2.92 | 4.31 | 3.87 | 2.0097 | -0.5785 | 0.3151 | 1.5985 | 1.1911 | 0.9072 | 1.6261 | 0.4929 |
| 24 | 2.80 | 4.33 | 4.56 | 3.96 | 3.03 | 0.2037 | 1.6171 | 1.8316 | 1.2742 | 0.4169 | 1.0687 | 1.7407 | 0.5568 |
| 25 | 6.56 | 3.82 | 2.18 | 3.95 | 2.84 | 3.7604 | 1.1450 | -0.3824 | 1.2650 | 0.2409 | 1.2058 | 1.8840 | 0.6308 |
